# Causes of death in women with breast cancer: a risks and rates study on a population-based cohort

**DOI:** 10.3389/fonc.2023.1270877

**Published:** 2023-11-01

**Authors:** Paolo Contiero, Roberto Boffi, Alessandro Borgini, Sabrina Fabiano, Andrea Tittarelli, Michael Mian, Fabio Vittadello, Susi Epifani, Antonino Ardizzone, Claudia Cirilli, Lorenza Boschetti, Stefano Marguati, Giuseppe Cascone, Rosario Tumino, Anna Clara Fanetti, Paola Giumelli, Giuseppa Candela, Tiziana Scuderi, Maurizio Castelli, Salvatore Bongiorno, Giulio Barigelletti, Viviana Perotti, Chiara Veronese, Fabio Turazza, Marina Crivaro, Giovanna Tagliabue, the MAPACA Working Group

**Affiliations:** ^1^Environmental Epidemiology Unit, Fondazione IRCCS Istituto Nazionale dei Tumori, Milan, Italy; ^2^Respiratory Disease Unit, Fondazione IRCCS Istituto Nazionale Tumori, Milan, Italy; ^3^Cancer Registry Unit, Fondazione IRCCS Istituto Nazionale dei Tumori, Milan, Italy; ^4^Innovation, Research and Teaching Service, Hospital of Bolzano (SABES-ASDAA), Teaching Hospital of the Paracelsus Medizinischen Privatuniversität (PMU), Bozen, Italy; ^5^Explora, Research and Statistical Analysis, Padova, Italy; ^6^Statistics and Epidemiology Unit, ASL Brindisi, Brindisi, Italy; ^7^Servizio di Epidemiologia e Comunicazione del Rischio-Unità Funzionale di Modena, Registro Tumori Regione, Modena, Italy; ^8^Pavia Cancer Registry, Public Health Agency of Pavia, Pavia, Italy; ^9^Ragusa Cancer Registry Unit, Azienda Sanitaria Provinciale di Ragusa (ASP), Ragusa, Italy; ^10^ATS della Montagna, S.S. Epidemiologia, Sondrio, Italy; ^11^Trapani and Agrigento Cancer Registry, Trapani, Italy; ^12^Dipartimento di Prevenzione Azienda USL Valle d’Aosta Loc, Quart, Italy; ^13^Cardiology Unit, Fondazione IRCCS Istituto Nazionale dei Tumori, Milan, Italy

**Keywords:** cardio-oncology, geographic variation, breast cancer, cardiovascular disease, respiratory disease, cause-specific death, competing-risk model, population-based cancer registries

## Abstract

**Introduction:**

The increasing survival of patients with breast cancer has prompted the assessment of mortality due to all causes of death in these patients. We estimated the absolute risks of death from different causes, useful for health-care planning and clinical prediction, as well as cause-specific hazards, useful for hypothesis generation on etiology and risk factors.

**Materials and methods:**

Using data from population-based cancer registries we performed a retrospective study on a cohort of women diagnosed with primary breast cancer. We carried out a competing-cause analysis computing cumulative incidence functions (CIFs) and cause-specific hazards (CSHs) in the whole cohort, separately by age, stage and registry area.

**Results:**

The study cohort comprised 12,742 women followed up for six years. Breast cancer showed the highest CIF, 13.71%, and cardiovascular disease was the second leading cause of death with a CIF of 3.60%. The contribution of breast cancer deaths to the CIF for all causes varied widely by age class: 89.25% in women diagnosed at age <50 years, 72.94% in women diagnosed at age 50–69 and 48.25% in women diagnosed at age ≥70. Greater CIF variations were observed according to stage: the contribution of causes other than breast cancer to CIF for all causes was 73.4% in women with stage I disease, 42.9% in stage II–III and only 13.2% in stage IV. CSH computation revealed temporal variations: in women diagnosed at age ≥70 the CSH for breast cancer was equaled by that for cardiovascular disease and “other diseases” in the sixth year following diagnosis, and an early peak for breast cancer was identified in the first year following diagnosis. Among women aged 50–69 we identified an early peak for breast cancer followed by a further peak near the second year of follow-up. Comparison by geographic area highlighted conspicuous variations: the highest CIF for cardiovascular disease was more than 70% higher than the lowest, while for breast cancer the highest CIF doubled the lowest.

**Conclusion:**

The integrated interpretation of absolute risks and hazards suggests the need for multidisciplinary surveillance and prevention using community-based, holistic and well-coordinated survivorship care models.

## 1 Introduction

In 2020 an estimated 2.26 million new cases of breast cancer were diagnosed across the globe (1). For women diagnosed during 2010–2014, the five-year survival for breast cancer was reported to range from 89.5% in Australia and 90.2% in the USA to 66.1% in India (2). The combination of high incidence and high survival has led to an increased interest in non-breast-cancer deaths following a breast cancer diagnosis. Cardiovascular disease (CVD) is an important cause of death following breast cancer: 1.6% to 10.4% of all women with breast cancer died of CVD (3), not only due to the high prevalence of CVD in the general population but also because of the overlapping risk factors between breast cancer and CVD and the adverse cardiovascular effects of cancer treatments.

A systematic review (3) showed that only a limited number of studies investigated the risk of CVD mortality following breast cancer, and these studies were heterogeneous in design, study populations and study periods and selection by age and stage of the women included in the analysis (4–11). Specific causes of cancer-related death other than breast cancer included ovarian and endometrial cancer, while pulmonary and gastrointestinal diseases were prominent among non-cancer causes (12–14).

The analysis of cause-specific deaths requires specific attention because of the competition between different causes of death. In breast cancer patients the mortality from a specific cause of death, for example CVD, is strongly influenced by breast cancer mortality, as the occurrence of breast cancer death precludes the possibility for a person to die from other diseases.

The analysis of competing mortality is useful in two main settings. The first is clinical resource allocation and predictive research; in this case the need is to estimate the absolute risk of dying in patients with a specific disease. The second is the setting of etiology studies, where it is important to study the instantaneous rate of occurrence of the event of interest in subjects still at risk of the event in every instant (hazard). The recommendation from the literature is to use cumulative incidence functions (CIFs) and risk ratios for the estimation of absolute risks, and cause-specific hazards (CSHs) and cause-specific hazard ratios (CSHRs) for the investigation of instantaneous rates (15–20). CIF describes the proportion of patients with a certain event over the course of time, and in the simplest case refers to the number of individuals initially enrolled in the study. For example, it could be the proportion of all patients in a breast cancer cohort who develop breast cancer recurrences in 10 years from diagnosis, e.g., 12% of the total of women initially included in the observation. CSH is a function of time and describes the instantaneous rate of occurrence of the event of interest in subjects who are still at risk of the event. For example, in a breast cancer cohort it is the rate at which patients develop recurrences during the second year of observation considering patients who survived until the beginning of the second year, for example one woman in 50 in one year. While many studies have investigated CIFs, very few have studied CSHs, and not a single paper has been published presenting the results of both metrics together. We therefore considered that a step forward in the knowledge and interpretation of the causes of death in women affected by breast cancer could be a study that analyzed hazards (CSHs) and absolute risks (CIFs) side by side, following the indications of leading authors according to whom this is the most rigorous scientific approach to evaluate competing risk data such as cause-specific deaths (21). By adopting this approach we were able to study the cumulative proportion of patients dying from a certain disease during the entire follow-up time along with the variations in the risk of death in different periods.

As a further study objective we decided to analyze the geographic variations of CIFs and CSHs. To our knowledge only the paper by Ho et al. (22) reported on this topic, concluding that other studies with both individual and county-level information are needed to inform public health interventions in this field.

We designed a competing-mortality study taking into account breast cancer deaths, other cancer-related deaths, CVD deaths, respiratory-disease deaths, and deaths from other causes than the above listed. We included all women with breast cancer regardless of age and disease stage. We decided to analyze the data in a relatively recent time span to be able to provide an up-to-date picture of the causes of death following breast cancer. We used a retrospective cohort design based on population-based registries, thereby avoiding any selection bias.

## 2 Materials and methods

### 2.1 Breast cancer cases

This was a retrospective study on a cohort of women diagnosed with primary breast cancer. The cases were archived in the following population-based cancer registries: Aosta Valley (2010–2013), Brindisi (2010–2013), Pavia (2010–2013), Modena (2014–2017), Ragusa and Caltanissetta (2010–2013), Sondrio (2010–2016), South Tyrol (2010–2016) and Trapani (2010–2013). Selection using the site code C50 and malignant epithelial morphology codes M8010–M8575 of the International Classification of Diseases for Oncology (ICD-O-3) (23) retrieved a total of 12,742 primary breast cancer cases diagnosed in the predetermined study period and meeting our selection criteria. All 12,742 cases were used in the analysis. Causes of death were categorized by the following ICD-10 codes (24): C50 (breast cancer), I00–I99 (CVD), C00–C97 except C50 (cancers other than breast), J00–J99 (respiratory diseases), and other causes than the ones listed. Disease stage was specified according to the sixth edition of the TNM classification of malignant tumors (25).

### 2.2 Statistical analysis

This study was based on the computation of two functions: CIF and CSH. CIF is generally used to estimate the absolute risk of the occurrence of an event of interest up to a follow-up time point *t* and refers to the number of individuals enrolled in the study. In a simplified case – observation without censoring – CIF at time *t* can be computed as the fraction D/N, where N is the number of individuals under study at time 0 and D is the number of persons who die or develop a disease in a specified time period, from 0 to *t*. CSH estimates the instantaneous probability at time *t* of an event, considering as denominator the population surviving up to time point *t*, thereby providing a picture of the instantaneous modifications in the risk under study. While CIF refers to the initial number of patients, CSH refers to patients surviving at time *t*. The CSH analysis results are useful to intercept variations in rates, taking into account at every time point the number of people surviving up to that moment and thereby making it possible to analyze determinants in the causes of disease development or progression rates. In survival analysis, risk takes the denomination of CIF and rate is substituted by CSH. We estimated CIFs and CSHs, taking all cases of the cohort together and separately by registry to investigate the extent of geographic variation. We also analyzed CIFs and CSHs separately by age (0–50, 50–69 and ≥70 years), stage (local, regional and metastatic) and registry. Differences in CSH were determined by log-rank test and those in CIF with Gray’s test (26). We used cubic splines to estimate CSH curves, exploring possible changes over time since the breast cancer diagnosis. We ran multivariate Cox proportional hazards models to estimate CSHRs with 95% confidence intervals (CI) of cause-specific deaths to analyze differences by registry, introducing into the model a variable for each of the eight cancer registries under analysis and stratifying the model for age as a potential confounding factor. Time to event or end of follow-up was calculated from the date of diagnosis. The hazard proportionality was tested by analysis of scaled Schoenfeld residuals. To explore the effect of competing causes of death on CIFs we estimated sub-distribution hazard ratios (SHRs) with the Fine-Gray model, which adjusts for the influence of other causes of death that may prevent cause-specific deaths from being observed. The analyses were performed according to the methods described in the competing risk literature (15–21), using the R statistical package (version 4.2.2) and the add-on packages Epi, cmprsk, crr-addson and splines (27–29). Differences were considered significant at *P*<0.05.

## 3 Results

### 3.1 Cohort characteristics

The median age at diagnosis of the cohort of 12,742 patients was 63 years (interquartile range 51–74). Stage information was available for 9131 (71.66%) women; the breakdown by stage I, II–III (grouped together for the study) and IV was 4024 (31.58%), 4418 (34.67%) and 689 (5.41%), respectively. A total of 2760 women died during the first six years, 1630 (12.79% of cohort, 59.06% of deaths) of breast cancer, 288 (2.3% of cohort, 10.43% of deaths) of cancers other than breast, 415 (3.2% of cohort, 15.04% of deaths) of CVD, 76 (0.59% of cohort, 2.75% of deaths) of respiratory diseases, and 351 (2.75% of cohort, 12.72% of deaths) of causes other than the above listed.

### 3.2 Analysis of cumulative incidence

**Figure 1:**
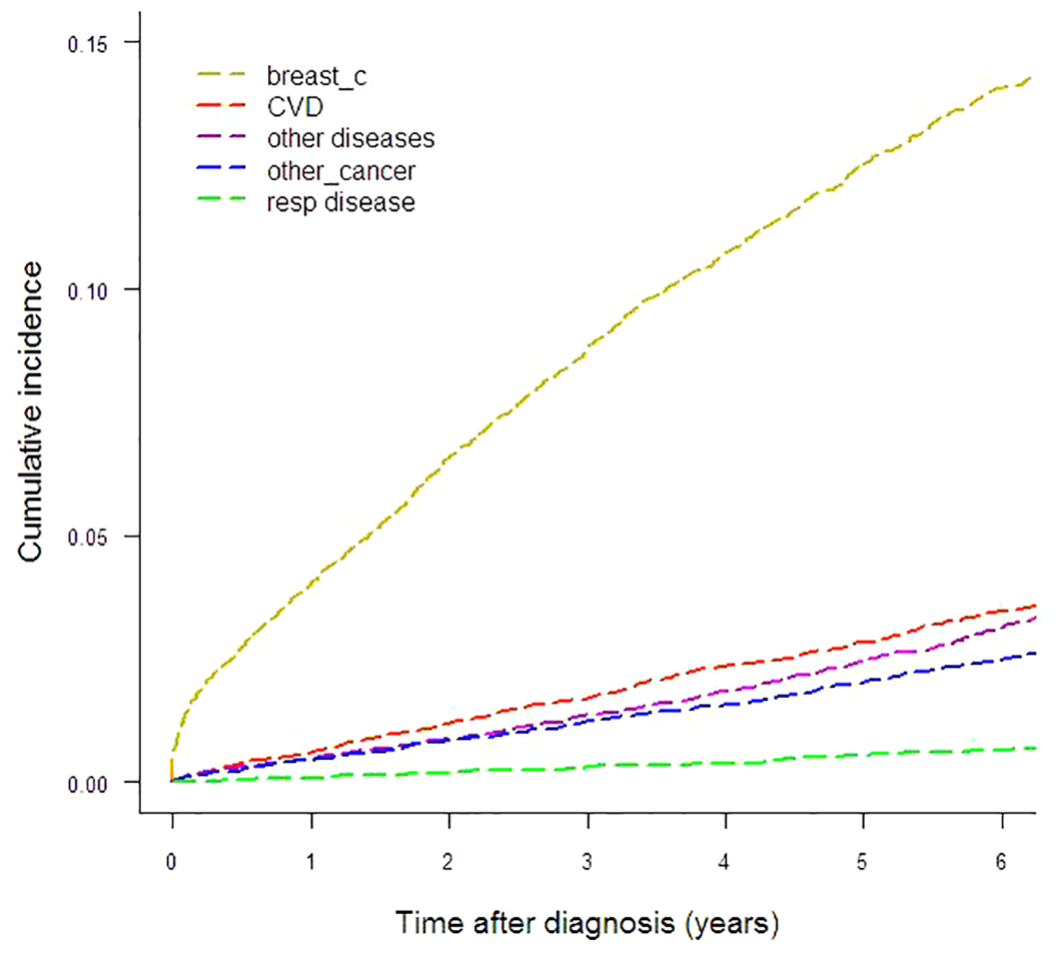
Cumulative incidence of mortality by different causes-of-death groups.

To illustrate the absolute risk of mortality in this breast cancer cohort, **Figure 1** and **Table 1** show the CIFs (%) for every cause under analysis by the year from diagnosis to six years of follow-up. Breast cancer presented the highest CIF (13.71%) and CVD ranked second (3.60%). CIF amounted to 3.10% for other diseases, 2.50% for other cancers and 0.66% for respiratory diseases. For CVD the most represented ICD-10 categories were cerebrovascular disease (n=117), chronic ischemic heart disease (n=61), hypertensive disease (n=58) and acute myocardial infarction (n=35). The main contributors to the “other diseases” category of deaths were diseases of the digestive system (n=55), mental and behavioral disorders (n=34), certain infectious and parasitic diseases (n=31), endocrine, nutritional and metabolic diseases (n=30, of which n=25 diabetes mellitus) and diseases of the nervous system (n=29). Malignant neoplasms of the bronchus and lung (n=35), colon (n=34), pancreas (n=27), liver and intrahepatic bile ducts (n=26), stomach (n=21) and ovary (n=16), multiple myeloma and malignant plasma cell neoplasm (n=11) were the largest contributing causes of death for the “other cancers” category. Among respiratory disease deaths pneumonia (n=37) was the most common cause.

**Table 1:** Cumulative incidence of cause-specific deaths by years of follow-up for all ages and by age class.

Cumulative incidence (%)									
Deaths	1y		2y		3y	4y	5y	6y	95% CI at 6y
Cardiovascular disease									
All ages	0.66		1.29		1.78	2.43	2.91	3.60	3.26-3.95
<50	0.00		0.00		0.04	0.04	0.08	0.08	0.02-0.28
50-69	0.16		0.20		0.33	0.53	0.62	0.76	0.54-1.04
≥70	1.66		3.39		4.60	6.17	7.39	9.17	8.30-10.08
Respiratory disease									
All ages	0.10		0.24		0.32	0.39	0.56	0.66	0.53-0.82
<50	0.00		0.00		0.00	0.00	0.04	0.04	0.01-0.24
50-69	0.00		0.02		0.02	0.04	0.04	0.04	0.01-0.13
≥70	0.29		0.64		0.89	1.06	1.51	1.79	1.42-2.24
Breast cancer									
All ages	3.84		6.31		8.54	10.50	12.29	13.71	13.09-14.34
<50	1.18		2.76		4.59	6.13	7.92	9.22	8.11-10.42
50-70	1.88		3.71		5.54	7.25	8.63	9.81	9.00-10.66
≥70	7.82		11.61		14.54	17.08	19.37	21.13	19.90-22.38
Other cancers									
All ages	0.48		0.87		1.28	1.59	2.05	2.50	2.23-2.81
<50	0.11		0.11		0.19	0.23	0.27	0.42	0.21-0.77
50-69	0.16		0.42		0.72	0.86	1.21	1.57	1.24-1.95
≥70	1.09		1.88		2.62	3.29	4.14	4.89	4.26-5.59
Other diseases									
All ages	0.47		0.92		1.38	1.87	2.47	3.10	2.79-3.43
<50	0.07		0.18		0.30	0.37	0.46	0.57	0.33-0.94
50-69	0.13		0.27		0.48	0.66	1.01	1.27	0.98-1.62
≥70	1.13		2.15		3.14	4.24	5.44	6.85	6.09-7.67
All causes									
All ages	5.55		9.64		13.31	16.79	20.29	23.57	22.8-24.35
<50	1.36		3.05		5.12	6.77	8.77	10.33	9.16-11.59
50-69	2.33		4.62		7.09	9.34	11.51	13.45	12.51-14.42
≥70	11.98		19.68		25.79	31.85	37.85	43.83	42.28-45.38

**Figure 2:**
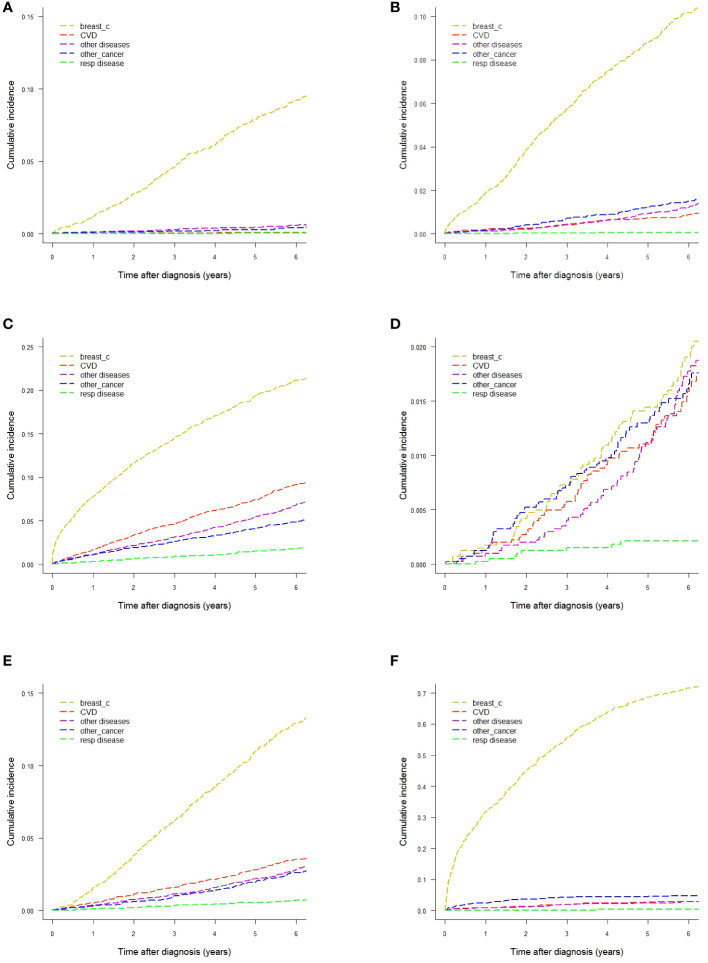
Cumulative incidence of mortality by different causes-of-death groups. **(A)** Age <50 years. **(B)** Age 50−69 years. **(C)** Age ≥70 years. **(D)** Women with stage I breast cancer. **(E)** Women with stage II−III breast cancer. **(F)** Women with stage IV breast cancer.

Gray’s test reported statistically significant differences of CIFs by age and stage for all five causes-of-death groups. This finding led us to explore CIFs separately for age and stage. CIFs by age are presented in **Table 1** and **Figures 2A–C** and CIFs by stage in **Table S1** (**Additional file 1**) and **Figures 2D–F**. The proportion for every specified cause of death with respect to the total CIF (all causes) showed variations according to age. In women diagnosed at age <50 years the CIF for breast cancer mortality was predominant, accounting for 89.25% of the total CIF (resulting from the ratio between the two CIFs, 9.22% and 10.33%, respectively). Likewise, the CIF for CVD accounted for only 0.77% of the total CIF. In women diagnosed at age 50–69, the CIF for breast cancer mortality was 72.94% and the CIF for CVD mortality was 7.36% of the total CIF, a marked increase with respect to the CIF in the <50-year age class. For women diagnosed at age ≥70 the CIF for breast cancer mortality diminished with respect to that of the younger age classes, accounting for only 48.25% of the total CIF, while the CIF for CVD mortality increased, accounting for 20.92% of the total CIF. Also the CIFs for other diseases varied according to age: the CIFs for death from respiratory diseases went from 0.66% (age <50) to 1.79% (age ≥70), for death from other cancers from 0.42% (age <50) to 4.89% (age ≥70), and for death from other diseases from 0.57% (age <50) to 6.85% (age ≥70). The CIFs for each disease group were markedly different in each stage group. The CIF for CVD mortality at six years from diagnosis was similar to that of breast cancer (1.59% and 1.90%, respectively) in women with stage I disease, while in stage II–III the CIF for breast cancer mortality was much higher (12.90%) than that for CVD mortality (3.54%). In women with stage IV disease breast cancer became by far the leading cause of death (71.64%), while the CIF for CVD deaths was 2.94%. The CIF for causes of death other than breast cancer accounted for 73.4% of the total CIF in women with stage I disease, while in stage II–III it was 42.9% and in stage IV only 13.2%.

We also performed specific analyses by different combinations of the staging variables T (primary tumor), N (regional lymph nodes) and M (distant metastases), and age (data not shown). The most interesting result was that in older women (≥70 years at diagnosis) with small-size cancers (T1) and no metastases at presentation the CIF for CVD mortality at six years from diagnosis was higher than that for breast cancer, 6.03% versus 5.68%, respectively.

### 3.3 Analysis of cause-specific hazards

**Figure 3:**
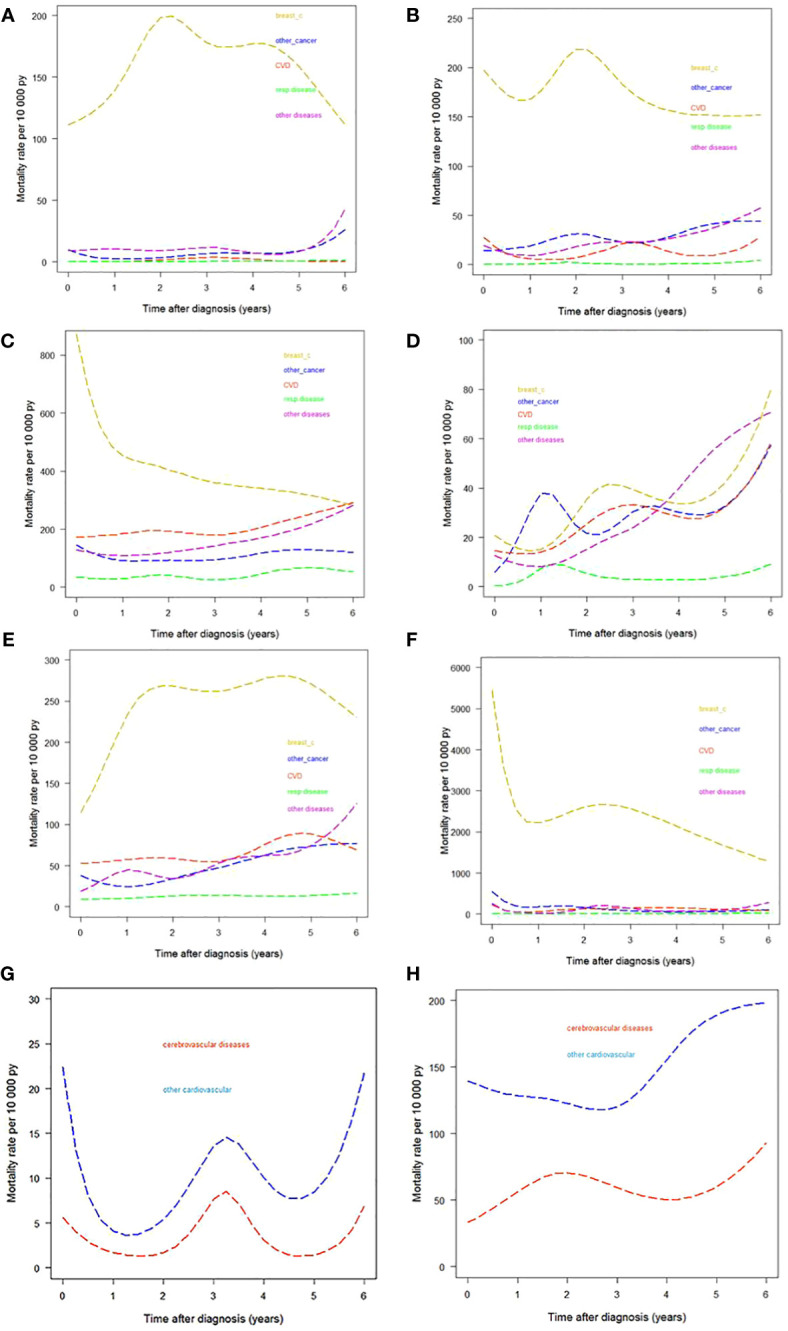
Cause-specific hazards by different causes-of-death groups. **(A)** Age <50 years. **(B)** Age 50−69 years. **(C)** Age ≥70 years. **(D)** Women with stage I breast cancer. **(E)** Women with stage II−III breast cancer. **(F)** Women with stage IV breast cancer. **(G)** CVD divided into cerebrovascular and other CVDs in women diagnosed at age 50–69. **(H)** CVD divided into cerebrovascular and other CVDs in women diagnosed at age ≥70. py, person years.

**Table 2** and **Figure 3** describe how CSHs in women with a breast cancer diagnosis varied by time since diagnosis for different age and stage groups. We observed a peak in breast cancer CSH in women diagnosed at age <50 (**Figure 3A**) occurring two to three years since the diagnosis. The CSH for CVD was very low during the whole observation period of six years. Among women diagnosed at age 50–69 (**Figure 3B**), we identified an early peak for breast cancer followed by a further peak between the second and third years of follow-up, almost at the same time as the peak observed in younger women. We identified a peak in the CSH of CVD in the first year after breast cancer diagnosis. The CSH for CVD ranked second near the diagnosis, while near the end of the follow-up period the CSH for the diagnostic group of other diseases ranked second. Among women diagnosed at age ≥70 (**Figure 3C**) we identified a strong early peak (first year after diagnosis) for breast cancer. Interestingly, by the sixth year following diagnosis the CSH for breast cancer deaths was matched by those for death from CVD and from the “other diseases” group.

**Table 2:** Cause-specific hazards by years of follow-up for all ages and by age class.

Cause-specific hazards per 10,000 person years							
Deaths	1y	2y	3y	4y	5y	6y	95% CI at 6y
Cardiovascular disease							
All ages	65.18	68.21	56.68	75.46	58.87	87.75	68.42-112.55
<50	0.00	0.00	3.89	0.00	4.78	0.00	NA
50-69	16.65	3.78	13.75	21.68	9.76	14.58	6.07-35.03
≥70	170.33	205.61	159.18	218.96	186.01	302.01	232.96-391.54
Respiratory disease							
All ages	9.78	14.50	9.90	8.05	20.78	11.32	5.66-22.64
<50	0.00	0.00	0.00	0.00	4.78	0.00	NA
50-69	0.00	1.89	0.00	2.17	0.00	0.00	NA
≥70	28.79	42.18	31.84	23.58	68.74	42.39	21.20-84.76
Breast cancer							
All ages	351.18	267.74	252.81	230.40	219.31	179.75	151.06-213.90
<50	111.13	162.26	190.81	165.32	196.05	137.21	91.96-204.70
50-69	186.86	189.24	194.43	186.49	153.69	137.07	102.99-182.44
≥ 70	719.71	450.76	384.93	350.34	347.75	296.71	228.34-385.56
Other cancers							
All ages	47.26	42.63	45.88	36.22	56.56	58.03	42.73-78.81
<50	7.41	0.00	7.79	4.24	4.78	17.15	5.53-53.18
50-69	14.80	26.49	31.42	15.18	39.03	40.83	24.18-68.94
≥70	115.15	94.90	95.51	94.32	129.40	127.16	85.23-189.72
Other causes							
All ages	44.81	48.60	52.18	57.35	72.72	79.26	61.00-102.99
<50	7.41	11.32	11.68	8.48	9.56	11.43	2.86-45.72
50-69	11.10	15.14	21.60	19.52	39.03	29.16	15.69-54.20
≥70	112.75	121.26	127.34	154.96	181.96	233.13	173.49-313.28
All causes							
All ages	518.21	441.69	417.45	407.48	428.23	416.11	371.17-466.51
<50	125.94	173.58	214.17	178.04	219.96	165.79	115.21-238.58
50-69	229.42	236.55	261.20	245.03	241.51	221.65	177.02-277.53
≥70	1146.74	914.70	798.79	842.17	913.86	1001.41	868.35-1154.86

CSH estimates by stage are presented in **Figures 3D−F**. The pattern of the diseases under analysis varied greatly by stage. In women with stage I breast cancer the contribution of breast cancer deaths was comparable to that of the group of “other diseases” at six years from diagnosis. We performed a further analysis splitting CVD into cerebrovascular and other cardiovascular diseases for women diagnosed at age 50–69 and at age ≥70. (We excluded women <50 years because of the low CVD CSH in this age class.) The results are shown in **Figures 3G, H**. Differences by age were as follows: in women diagnosed at age 50–69, the CSH for other cardiovascular diseases peaked in the first year after diagnosis and both CVD subgroups showed a peak at the third year of follow-up and an increase in the last years of observation; in women diagnosed at age ≥70 the CSH for cerebrovascular disease showed a peak at the end of the first year after diagnosis, while the CSHs for the two CVD subgroups increased from the fifth year onward.

### 3.4 Comparison of CIFs and CSHs between registries

Gray’s test for subdistribution hazards revealed statistically significant differences in CIF by registry for all five causes-of-death groups, prompting us to compute CIFs by registry (**Supplementary Table S2**). Interestingly, the highest CIF value among registries for all-cause deaths (26.49%) was almost 25% higher than the lowest (21.22%). The two highest CIFs for CVD (4.24% and 4.19%) were more than 70% higher than the lowest (2.43%). The highest CIF at six years for breast cancer (18.50%) was 2.2 times the lowest (8.16%). The CIFs for other cancers ranged from 1.46% to 3.60% and those for respiratory diseases from 0.14% to 2.28%. CIFs for the “other diseases” category ranged from 1.73% to 6.63%.

Similarly to the analysis of CIFs, computation of CSHs at the sixth year for CVD by registry (**Supplementary Table S3**) showed highest values (143.15 and 132.47) that were markedly higher than the lowest (21.70 and 68.18). For breast cancer the highest CSH was 294.54 and the lowest 90.90. For the category “other cancers” the highest CSH was 66.11 and the lowest 33.66. The highest CSH for respiratory diseases was 67.33, while the CSH for this disease category was 0 in three registries. Fine-Gray SHR models were run with the inclusion of a covariate for age and taking the cancer registry of Trapani as the reference (**Supplementary Table S4**). The risk excesses identified by the computation of SHRs for CVD, with highest values of 1.65 (1.06–2.58) and 1.63 (1.01–2.62), match the comparison of CIFs by registry, where the highest CIFs were more than 70% higher than the lowest. Computation of the other SHRs confirms the results obtained in the comparison between CIFs. Cox models were run including a covariate for age (**Supplementary Table S5**) and taking the cancer registry of Trapani as the reference. The highest CSHRs for CVD, 1.69 (1.08–2.63) and 1.64 (1.02–2.63), confirm the differences in CSHs by registry, where the highest CSHs were more than 70% higher than the lowest.

## 4 Discussion

This study was designed to give a complete epidemiologic picture of the event dynamics of causes of death in a competing-mortality setting analyzing absolute risks (CIFs) and hazards (CSHs) side by side. Important CIF variations for different causes of death were identified by age class and stage, and the relevance of causes of death other than breast cancer was demonstrated. Analysis of CSHs revealed marked variations during the follow-up period, identifying peaks that were useful to understand the development of all diseases that may affect women with a diagnosis of breast cancer. This information completes the analysis of CIFs, because it shows that diseases other than breast cancer that contribute significantly to the absolute risk of dying (CIF) do not act uniformly during the follow-up period but are more likely to present in specific moments in time.

These results support our hypothesis that side-by-side computation of CIFs and CSHs may provide a more complete epidemiologic picture of causes of death following a breast cancer diagnosis. With regard to CIF (a measure of absolute risks), our results pointed in the same direction as studies using similar metrics. Clèries et al. (14), despite differences in the structure of their study with respect to ours, obtained similar results. For example, in women with stage I breast cancer we both found that the non-cancer mortality surpassed breast cancer deaths after some years from diagnosis. We are also in agreement with the main result of the study by Afifi et al. (12), according to which causes of death other that breast cancer (mainly heart and cerebrovascular diseases) account for a significant number of deaths among patients with a breast cancer diagnosis.

The computation of absolute risks allowed us to identify CVD as the second leading cause of death over a six-year follow-up with a CIF of 3.60%, amounting to 15.27% of CIFs for all causes. These results are similar to what Gernaat et al. reported in their review (3), according to which deaths from CVD ranged from 1.6% to 10.4%. Comparing our results with those of the studies included in the review is difficult due to differences in the composition of the cohorts; for example, the studies by Hooning et al. (6) and Solanki et al. (30) selected women with stage I–III breast cancer, while our cohort consisted of women with infiltrating breast cancer regardless of stage. In addition, the studies included in the review were performed in earlier periods than ours. Nevertheless, all of them identified CVD as the leading cause of competing mortality with respect to breast cancer. The study by Abdel-Qadir et al. (11) on women with a diagnosis of early-stage breast cancer concluded that CVD death is an important competing risk in older women with early-stage breast cancer, which is in agreement with our results.

The other principal metric we computed was CSH, analyzing it over time since diagnosis. To our knowledge only a paper by Colzani et al. (31) reported similar CSH computations. Like Colzani et al. we observed a breast cancer CSH peak in women aged <50 and 50–69 two to three years from the breast cancer diagnosis, but we failed to see such a peak in women aged ≥70 years. This difference may be due to the different selection criteria of the Colzani study, which included only women aged ≤75 years without metastases at diagnosis. We identified a peak in CSH for CVD in women aged 50–69 years, while Colzani et al. observed a peak in the 65–74-year age class. Demicheli et al. (32) identified peaks similar to ours when analyzing disease progression in breast cancer.

The results of this paper may also be useful to generate hypotheses about disease progression following a breast cancer diagnosis. Breast cancer metastases have been shown to appear at variable intervals (33), and two hypotheses have been proposed to explain this. The first postulated uninterrupted tumor growth, while the second hypothesized tumor progression as a discontinuous process alternating states of dormancy followed by rapid growth. In our study we did not analyze metastatic spread but mortality, which depends also on treatments after the occurrence of metastases. However, some common inferences might be drawn. Although our study was not designed to test these two hypotheses on progression, the presence of multiple peaks in the time trend of some CSHs and the discontinuities we observed favor the dormancy hypothesis. To analyze which factors prompt the onset of tumor growth, *ad hoc* studies will be needed.

The differences we observed in CIFs and CSHs could be explored by taking into account exposure to different diets and environmental pollutants. However, these aspects are beyond the scope of this paper, which aims to present the first population-based analysis in Europe using CSHs and CIFs. Further analysis will be performed on the dataset to explain risk factors.

A further aim of our study was to investigate the geographic variations of CIF and CSH. CIFs and CSHs showed remarkable differences. For example, the CIF for CVD in the cancer registry with the highest value was more than 70% higher than the CIF for CVD in the cancer registry with the lowest value. To our knowledge the only study that reported variations in cause-specific deaths according to geographic region was the one by Ho et al. (22). They drew similar conclusions to ours, identifying an association of geographic factors at breast cancer diagnosis with an increased CVD mortality risk. Since no studies have been published about the geographic distribution of causes of death other than breast cancer and CVD following a breast cancer diagnosis, we cannot compare our study to others in this respect. From a methodological point of view, this study confirms the notion, shared with many experts on prevention, that cancer is a disease showing marked geographical heterogeneity. The implications are relevant from a public health point of view because they highlight the need for nuanced and geographically specific rather than generalized policies, a belief we share with other authors (34). In the area we observed, further investigations are needed to address, for example, the death hazards for CDV in South Tyrol and Ragusa-Caltanissetta with respect to those of the Trapani province, and the breast cancer death rates of Trapani with respect to those of other provinces.

A limitation of our study is the presence of “R99” (Ill-defined and unknown cause of mortality) (n=115) among the ICD-10 mortality codes; however, its percentage, 0.9% of the whole cohort, can hardly be considered to invalidate the results. The analysis of cause-specific mortality may be challenging because of the possible misclassification of causes of death. A study in Italy (35) on a population-based cohort re-evaluated causes of death as classified by death certificates, revealing only a slight overestimate of deaths attributed to breast cancer. A study in Belgium (36) reported a fair agreement (84.7%) between death certificates and medical files for women with a breast cancer diagnosis treated at University Hospitals Leuven. A paper by Schaffar et al. (37) reported a high overall agreement comparing the causes of death by official death certificates and those revised by personnel of the population-based cancer registry of Genève (Switzerland). Considering the results of these studies we can assume that the bias related to misclassification of specific causes of death in our study was minimal.

A further limitation of the study is that information on breast cancer stage was available for only 71.7% of cases.

We decided to observe the follow-up until the sixth year from diagnosis in order to use the most up-to-date data available, which allowed us to describe the most recent epidemiology of the causes of death. However, this choice precluded us from observing what happened from the sixth year onward.

A major strength of our study is that for the first time, to our knowledge, CIFs and CSHs were analyzed side by side, enabling us to measure absolute risks and rate variations at the same time. A further strength is the population-based design, which allowed us to describe what happened in the general population of a vast area, taking into account women at whichever age and whichever breast cancer stage. This prevented the introduction of selection bias and enhanced the generalizability of our results. Likewise, we analyzed for the first time in Europe and to our knowledge the second time worldwide geographic variations in the risks and rates of cause-specific mortality. Geographic variations in risks are very important to identify because they may point to a different distribution of modifiable risk factors. The availability of such information may prompt changes in the clinical follow-up of patients and indicate regional differences in the pathologic features of breast cancer. It can also direct etiologic research and be used in the planning of geographically tailored preventive and clinical strategies and resource allocation.

The results of this study also suggest considerations in the setting of tertiary prevention because of the weight of non-breast-cancer deaths among women with a breast cancer diagnosis. The first two causes of death we observed were breast cancer and CVD. The risk of disease progression in breast cancer is associated with modifiable factors such as cigarette smoking, obesity, metabolic syndrome and diabetes, high blood pressure and a sedentary lifestyle, with some studies also identifying a link with atmospheric particulate matter exposure (38–40). Breast cancer progression shares these risk factors with CVD and other cancers such as colorectal cancer, suggesting common prevention pathways. For example, in the setting of physical activity and exercise it has been proposed to extend the Cardiac Rehabilitation model to a Cardio-Oncology Rehabilitation (CORE) model (40), defined as “an exercise-based multicomponent intervention to improve the care and prognosis of a patient’s cancer”. The model encourages the use of existing, ready-to-use resources, including a network of professionals dedicated to cardiac rehabilitation.

The need to take into account causes of death other than breast cancer in women with a breast cancer diagnosis is also related to survivorship care models. Some authors (41–44) proposed the use of innovative models for effective clinical governance of survivorship care based on a possible role of the “community oncologist”, defined as a trained health professional acting as a link between hospital specialists, who are frequently overburdened, and general practitioners. The results of our study encourage the application of new methods for the management of survivorship.

## 5 Conclusions

The results of our competing-cause analysis show that causes of death other than breast cancer are important in women with a diagnosis of breast cancer, especially women aged ≥50 years and those with stage I–III cancer, and that causes of death vary with time and also between registry areas. These results underscore the need for oncologists to balance the types and intensity of breast cancer treatment, taking into account possible cardiovascular and other side effects and the application of differential follow-up pathways. Furthermore, the observed geographic differences warrant research into the association of such differences with risk factors. The integrated interpretation of absolute risks and hazards highlights the necessity of surveillance and prevention by a multidisciplinary approach, and the need for community-based, holistic and well-coordinated survivorship care models.

## Data availability statement

The datasets presented in this article are not readily available because In respect to privacy legislation the dataset could not be accessed. Requests to access the datasets should be directed to alessandro.borgini@istitutotumori.mi.it.

## Ethics statement

The study was approved by the Ethics Committee of Fondazione IRCCS Istituto Nazionale dei Tumori, Milan, Italy. Record number 155/19.

## Author contributions

AB: Data curation, Investigation, Validation, Writing – original draft. PC: Conceptualization, Formal Analysis, Methodology, Supervision, Writing – original draft. RB: Conceptualization, Writing – review & editing. SF: Data curation, Investigation, Validation, Writing – review & editing. AT: Data curation, Investigation, Writing – original draft. MM: Data curation, Validation, Writing – review & editing. FV: Data curation, Validation, Writing – review & editing. SE: Data curation, Validation, Writing – review & editing. AA: Data curation, Validation, Writing – review & editing. CC: Data curation, Validation, Writing – review & editing. LB: Data curation, Validation, Writing – review & editing. SM: Data curation, Validation, Writing – review & editing. GCas: Data curation, Validation, Writing – review & editing. RT: Data curation, Validation, Writing – review & editing. AF: Data curation, Validation, Writing – review & editing. PG: Data curation, Validation, Writing – review & editing. GCan: Data curation, Validation, Writing – review & editing. TS: Data curation, Validation, Writing – review & editing. MC: Data curation, Validation, Writing – review & editing. SB: Data curation, Validation, Writing – review & editing. GB: Investigation, Software, Writing – review & editing. VP: Validation, Writing – review & editing. CV: Validation, Writing – review & editing. FT: Validation, Writing – review & editing. MC: Validation, Writing – review & editing. GT: Conceptualization, Methodology, Validation, Writing – review & editing.

## Group members of the MA.PA.CA Working Group (WG)

Emma Cozzi^1^, Lucia Elena De Lorenzis^1^, Fernanda Lucia Lotti^1^, Maria Carmela Pagliara^1^, Ausilia Sferrazza^2^, Spata Eugenia^2^, Concetta Rollo^2^, Ivan Cometti^3^, Mariuccia Sala^3^, Gianfabrizio Ferrari^3^

^1^Statistics and Epidemiology Unit, ASL Brindisi, Brindisi, Italy

^2^Ragusa Cancer Registry Unit, Azienda Sanitaria Provinciale di Ragusa (ASP), Italy

^3^ATS della Montagna, S.S. Epidemiologia, Sondrio, Italy
