# Supplementary material for: Causes of death in women with breast cancer: a risks and rates study on a population-based cohort
Source: Front Oncol. 2023 Nov 1;13:1270877. doi: 10.3389/fonc.2023.1270877 (PMC10646497; doi:10.3389/fonc.2023.1270877)
Supplement: Supplementary file 1 [file Table_1.docx]

**Table S1** Cumulative incidence (CIF) of cause-specific deaths by years of follow-up, arranged by breast cancer stage

| **Cumulative incidence (%)** | | | | | | | | |
| --- | --- | --- | --- | --- | --- | --- | --- | --- |
| **Deaths** | **1y** | **2y** | | **3y** | **4y** | **5y** | **6y** | **95% CI at 6y** |
| Breast cancer stage I | | | | | | | | |
| Breast cancer | 0.17 | | 0.42 | 0.73 | 1.09 | 1.45 | 1.90 | 1.47-2.42 |
| Cardiovascular | 0.15 | | 0.30 | 0.58 | 0.94 | 1.10 | 1.59 | 1.20-2.07 |
| Other cancer | 0.12 | | 0.52 | 0.73 | 0.98 | 1.30 | 1.66 | 1.27-2.14 |
| Other diseases | 0.10 | | 0.20 | 0.40 | 0.69 | 1.12 | 1.78 | 1.35-2.30 |
| Respiratory disease | 0.02 | | 0.12 | 0.15 | 0.15 | 0.21 | 0.21 | 0.10-0.41 |
| All causes | 0.70 | | 1.79 | 2.81 | 4.04 | 5.32 | 7.14 | 6.28-8.08 |
| Breast cancer stage II-III | | | | | | | | |
| Breast cancer | 1.59 | | 3.79 | 6.17 | 8.55 | 10.96 | 12.90 | 11.87-13.97 |
| Cardiovascular | 0.52 | | 1.13 | 1.57 | 2.15 | 2.81 | 3.54 | 2.99-4.16 |
| Other cancer | 0.36 | | 0.61 | 1.01 | 1.40 | 1.97 | 2.61 | 2.14-3.16 |
| Other causes | 0.32 | | 0.75 | 1.14 | 1.59 | 2.21 | 2.85 | 2.35-3.41 |
| Respiratory disease | 0.11 | | 0.18 | 0.34 | 0.44 | 0.55 | 0.72 | 0.49-1.03 |
| All causes | 2.90 | | 6.47 | 10.23 | 14.13 | 18.50 | 22.61 | 21.30-23.95 |
| Breast cancer stage IV | | | | | | | | |
| Breast cancer | 31.86 | | 45.14 | 55.69 | 63.95 | 68.58 | 71.64 | 67.90-75.03 |
| Cardiovascular | 0.87 | | 1.31 | 1.90 | 2.39 | 2.75 | 2.94 | 1.82-4.47 |
| Other cancer | 2.47 | | 3.64 | 4.23 | 4.39 | 4.57 | 4.77 | 3.33-6.58 |
| Other diseases | 0.87 | | 1.16 | 1.91 | 2.23 | 2.41 | 2.84 | 1.73-4.37 |
| Respiratory disease | 0.15 | | 0.15 | 0.15 | 0.31 | 0.31 | 0.31 | 0.06-1.08 |
| All causes | 36.22 | | 51.40 | 63.87 | 73.28 | 78.63 | 82.50 | 79.16-85.35 |

**Table S2** Cumulative incidence (CIF) of cause-specific deaths by years of follow-up, arranged by registry

| **Cumulative incidence (%)** | | | | | | | | | | | | |
| --- | --- | --- | --- | --- | --- | --- | --- | --- | --- | --- | --- | --- |
| **Deaths** | **1y** | **2y** | **3y** | | **4y** | | **5y** | | **6y** | | **95% CI at 6y** | |
| Breast cancer | | | | | | | | | | | | |
| South Tyrol | 3.00 | 5.23 | 7.45 | | 9.51 | | 11.29 | | 12.08 | | 10.76-13.48 | |
| Brindisi | 3.25 | 6.74 | 9.05 | | 10.77 | | 13.54 | | 14.79 | | 12.94-16.76 | |
| Modena | 2.53 | 4.07 | 5.43 | | 6.48 | | 7.35 | | 8.16 | | 6.81-9.66 | |
| Pavia | 5.24 | 7.84 | 10.18 | | 11.94 | | 13.38 | | 14.92 | | 13.48-16.44 | |
| Ragusa-Caltanissetta Caltanissetta | 4.91 | 7.47 | 10.17 | | 13.01 | | 14.91 | | 16.45 | | 14.60-18.39 | |
| Sondrio | 4.27 | 7.12 | 9.53 | | 11.50 | | 13.21 | | 14.64 | | 12.64-16.79 | |
| Trapani | 5.22 | 7.88 | 10.83 | | 13.60 | | 16.28 | | 18.50 | | 16.21-20.91 | |
| Aosta Valley | 2.58 | 6.25 | 8.42 | | 10.61 | | 11.05 | | 12.94 | | 10.00-16.25 | |
| Cardiovascular disease | | | | | | | | | | | | |
| South Tyrol | 0.91 | 1.65 | | 2.14 | | 2.71 | | 3.19 | | 4.24 | | 3.42-5.18 |
| Brindisi | 0.59 | 0.96 | | 1.56 | | 1.93 | | 2.53 | | 2.70 | | 1.93-3.68 |
| Modena | 0.63 | 1.15 | | 1.76 | | 2.49 | | 2.75 | | 3.20 | | 2.41-4.17 |
| Pavia | 0.67 | 1.52 | | 2.02 | | 2.78 | | 3.55 | | 4.19 | | 3.41-5.08 |
| Ragusa-Caltanissetta | 0.47 | 0.94 | | 1.28 | | 2.23 | | 2.70 | | 3.79 | | 2.89-4.86 |
| Sondrio | 0.62 | 1.25 | | 1.78 | | 2.32 | | 2.68 | | 3.35 | | 2.40-4.53 |
| Trapani | 0.37 | 1.10 | | 1.38 | | 1.65 | | 1.84 | | 2.43 | | 1.61-3.52 |
| Aosta Valley | 1.07 | 1.72 | | 2.16 | | 3.25 | | 3.47 | | 3.47 | | 2.07-5.44 |
| Other cancers | | | | | | | | | | | | |
| South Tyrol | 0.62 | 0.99 | | 1.44 | | 1.71 | | 2.08 | | 2.54 | | 1.93-3.29 |
| Brindisi | 0.30 | 0.74 | | 1.41 | | 1.71 | | 2.31 | | 2.72 | | 1.94-3.70 |
| Modena | 0.40 | 0.95 | | 1.15 | | 1.59 | | 1.96 | | 2.23 | | 1.58-3.06 |
| Pavia | 1.03 | 1.57 | | 2.20 | | 2.60 | | 3.10 | | 3.60 | | 2.88-4.43 |
| Ragusa-Caltanissetta | 0.00 | 0.27 | | 0.67 | | 1.01 | | 1.76 | | 2.19 | | 1.53-3.03 |
| Sondrio | 0.36 | 0.53 | | 0.62 | | 0.71 | | 1.25 | | 1.73 | | 1.08-2.64 |
| Trapani | 0.28 | 0.46 | | 0.74 | | 0.92 | | 1.01 | | 1.46 | | 0.85-2.35 |
| Aosta Valley | 0.43 | 0.65 | | 1.08 | | 1.30 | | 1.52 | | 1.83 | | 0.86-3.46 |
|  |  |  | |  | |  | |  | |  | |  |
| Other diseases | | | | | | | | | | | | |
| South Tyrol | 0.45 | 1.15 | | 1.44 | | 1.98 | | 2.47 | | 2.95 | | 2.28-3.76 |
| Brindisi | 0.59 | 0.96 | | 1.26 | | 1.56 | | 2.16 | | 3.03 | | 2.19-4.07 |
| Modena | 0.36 | 0.87 | | 1.63 | | 2.43 | | 3.68 | | 6.63 | | 4.94-8.65 |
| Pavia | 0.67 | 0.94 | | 1.57 | | 1.98 | | 2.47 | | 3.06 | | 2.40-3.84 |
| Ragusa-Caltanissetta | 0.47 | 0.61 | | 1.08 | | 1.69 | | 2.16 | | 2.39 | | 1.70-3.28 |
| Sondrio | 0.45 | 0.89 | | 1.34 | | 1.61 | | 2.32 | | 2.99 | | 2.10-4.12 |
| Trapani | 0.27 | 0.73 | | 0.83 | | 1.20 | | 1.49 | | 1.73 | | 1.06-2.67 |
| Aosta Valley | 0.43 | 1.29 | | 1.73 | | 2.17 | | 2.82 | | 2.82 | | 1.58-4.65 |
| Respiratory disease | | | | | | | | | | | | |
| South Tyrol | 0.17 | 0.33 | | 0.37 | | 0.42 | | 0.57 | | 0.71 | | 0.41-1.16 |
| Brindisi | 0.00 | 0.00 | | 0.07 | | 0.22 | | 0.30 | | 0.39 | | 0.15-0.87 |
| Modena | 0.12 | 0.32 | | 0.48 | | 0.59 | | 0.83 | | 0.99 | | 0.57-1.62 |
| Pavia | 0.13 | 0.22 | | 0.31 | | 0.36 | | 0.58 | | 0.72 | | 0.43-1.15 |
| Ragusa-Caltanissetta | 0.00 | 0.07 | | 0.07 | | 0.14 | | 0.14 | | 0.14 | | 0.03-0.47 |
| Sondrio | 0.09 | 0.36 | | 0.53 | | 0.53 | | 0.72 | | 0.72 | | 0.34-1.36 |
| Trapani | 0.00 | 0.09 | | 0.09 | | 0.09 | | 0.19 | | 0.19 | | 0.04-0.65 |
| Aosta Valley | 0.43 | 0.65 | | 0.86 | | 1.08 | | 1.74 | | 2.28 | | 1.17-4.03 |

**Table S3** Cause-specific hazards of death (CSH) by years of follow-up, arranged by registry

| **Cause-specific hazards per 10,000 person years** | | | | | | | |
| --- | --- | --- | --- | --- | --- | --- | --- |
| **Deaths** | **1y** | **2y** | **3y** | **4y** | **5y** | **6y** | **95% CI at 6y** |
| Breast cancer | | | | | | | |
| South Tyrol | 304.34 | 239.32 | 250.25 | 242.00 | 220.96 | 99.35 | 56-175 |
| Brindisi | 320.07 | 375.12 | 259.86 | 201.40 | 339.49 | 162.73 | \| 98-270 \|  \| \| --- \| --- \| |
| Modena | 247.30 | 163.77 | 150.27 | 119.62 | 101.37 | 90.90 | 34-242 |
| Pavia | 475.36 | 288.62 | 272.75 | 213.92 | 183.65 | 204.35 | 146-286 |
| Ragusa-Caltanissetta | 426.06 | 277.17 | 312.21 | 329.73 | 237.36 | 200.41 | 131-307 |
| Sondrio | 279.60 | 309.21 | 272.85 | 233.07 | 208.48 | 185.64 | 112-308 |
| Trapani | 496.03 | 290.78 | 334.56 | 328.18 | 329.42 | 294.54 | 202-510 |
| Aosta Valley | 266.23 | 400.61 | 273.24 | 234.70 | 54.79 | 235.65 | 112-494 |
| Cardiovascular disease | | | | | | | |
| South Tyrol | 88.77 | 79.77 | 55.61 | 68.39 | 58.49 | 132.47 | 81-216 |
| Brindisi | 60.97 | 39.91 | 67.06 | 43.78 | 73.40 | 21.70 | 5-87 |
| Modena | 64.87 | 54.59 | 68.30 | 81.85 | 30.41 | 68.18 | 22-211 |
| Pavia | 65.89 | 94.55 | 57.70 | 93.25 | 97.56 | 84.14 | 50-142 |
| Ragusa-Caltanissetta | 48.89 | 51.06 | 45.69 | 104.55 | 59.34 | 143.15 | 86-237 |
| Sondrio | 46.60 | 67.64 | 60.63 | 63.57 | 43.89 | 86.63 | 41-182 |
| Trapani | 38.16 | 80.21 | 31.37 | 32.82 | 23.53 | 73.64 | 31-177 |
| Aosta Valley | 110.93 | 70.70 | 49.68 | 130.39 | 27.40 | 90.40 | 29-280 |
| Other cancers | | | | | | | |
| South Tyrol | 59.18 | 39.89 | 50.98 | 31.57 | 45.49 | 57.95 | 28-122 |
| Brindisi | 30.48 | 47.89 | 75.44 | 35.03 | 73.40 | 54.24 | 23-130 |
| Modena | 40.54 | 58.79 | 22.77 | 50.37 | 40.55 | 45.45 | 11-182 |
| Pavia | 108.25 | 59.71 | 73.43 | 49.37 | 63.13 | 66.11 | 37-119 |
| Ragusa-Caltanissetta | 0.00 | 29.18 | 45.69 | 40.21 | 93.25 | 57.26 | 26-127 |
| Sondrio | 18.64 | 19.33 | 10.11 | 10.59 | 65.84 | 61.88 | 26-149 |
| Trapani | 28.62 | 20.05 | 31.37 | 21.88 | 11.76 | 58.91 | 22-157 |
| Aosta Valley | 44.37 | 23.57 | 49.68 | 26.08 | 27.40 | 33.66 | 5-239 |
|  |  |  |  |  |  |  |  |
| Other diseases | | | | | | | |
| South Tyrol | 42.27 | 75.34 | 32.44 | 63.13 | 58.49 | 57.95 | 28-122 |
| Brindisi | 60.97 | 39.91 | 33.53 | 35.03 | 73.40 | 108.49 | 58-202 |
| Modena | 32.43 | 54.59 | 81.97 | 88.14 | 141.92 | 318.16 | 188-537 |
| Pavia | 70.60 | 29.86 | 73.43 | 49.37 | 63.13 | 78.13 | 45-135 |
| Ragusa-Caltanissetta | 48.89 | 14.59 | 53.30 | 72.38 | 59.34 | 28.63 | 9-89 |
| Sondrio | 18.64 | 48.31 | 50.53 | 31.78 | 87.78 | 86.63 | 41-182 |
| Trapani | 28.62 | 50.13 | 10.46 | 43.76 | 35.29 | 29.45 | 7-118 |
| Aosta Valley | 44.37 | 94.26 | 49.68 | 52.16 | 82.19 | 60.00 | 15-241 |
| Respiratory disease | | | | | | | |
| South Tyrol | 12.68 | 17.73 | 4.63 | 5.26 | 19.50 | 16.56 | 4-66 |
| Brindisi | 0.00 | 0.00 | 8.38 | 17.51 | 9.18 | 10.85 | 2-77 |
| Modena | 12.16 | 21.00 | 18.21 | 12.59 | 30.41 | 22.73 | 3-161 |
| Pavia | 14.12 | 9.95 | 10.49 | 5.49 | 28.69 | 18.03 | 6-56 |
| Ragusa-Caltanissetta | 0.00 | 7.29 | 0.00 | 8.04 | 0.00 | 0.00 | NA  NA |
| Sondrio | 9.32 | 28.99 | 20.21 | 0.00 | 21.95 | 0.00 | NA |
| Trapani | 0.00 | 10.03 | 0.00 | 0.00 | 11.76 | 0.00 | NA |
| Aosta Valley | 44.37 | 23.57 | 24.84 | 26.08 | 82.19 | 67.33 | 17-269 |

**Table S4** Cause-specific hazard ratios (CSHR) by registry in women with a diagnosis of breast cancer

| **Hazard ratios (95% CI)** | | | | | |
| --- | --- | --- | --- | --- | --- |
| **Cancer registry** | **Cardiovascular** | **Respiratory** | **Breast cancer** | **Other cancers** | **Other diseases** |
| South Tyrol | 1.69 (1.08-2.63) | 3.45 (0.79-15.09) | 0.62 (0.51-0.74) | 1.73 (0.98-3.07) | 1.66 (0.98-2.80) |
| Brindisi | 1.30 (0.78-2.17) | 2.25 (0.44-11.56) | 0.81 (0.67-0.99) | 2.09 (1.14-3.82) | 1.90 (1.07-3.33) |
| Modena | 1.27 (0.80-2.03) | 4.58 (1.06-19.74) | 0.40 (0.33-0.49) | 1.50 (0.83-2.69) | 2.49 (1.48-4.16) |
| Pavia | 1.55 (1.00-2.42) | 3.27 (0.75-14.22) | 0.75 (0.63-0.90) | 2.35 (1.35-4.07) | 1.61 (0.96-2.70) |
| Ragusa-Caltanissetta | 1.64 (1.02-2.63) | 0.74 (0.10-5.26) | 0.89 (0.74-1.08) | 1.57 (0.85-2.89) | 1.43 (0.81-2.53) |
| Sondrio | 1.41 (0.85-2.35) | 3.81 (0.81-17.92) | 0.78 (0.63-0.96) | 1.19 (0.61-2.35) | 1.74 (0.98-3.09) |
| Aosta Valley | 1.39 (0.74-2.62) | 10.76 (2.37-49.05) | 0.64 (0.48-0.86) | 1.18 (0.50-2.79) | 1.60 (0.78-3.27) |
| Trapani | 1 | 1 | 1 | 1 | 1 |

*Cox model stratified by age class

**Table S5** Subdistribution hazard ratios (SHR) by cause of death, arranged by registry, in women with a diagnosis of breast cancer

| **Subdistribution hazard ratios** | | | | | |
| --- | --- | --- | --- | --- | --- |
| **Cancer registry** | **Cardiovascular** | **Respiratory** | **Breast cancer** | **Other cancers** | **Other diseases** |
| South Tyrol | 1.65 (1.06-2.58) | 3.50 ( 0.80-15.30) | 0.64 (0.53-0.77) | 1.72 (0.97-3.04) | 1.66 (0.98-2.80) |
| Brindisi | 1.28 (0.76-2.14) | 2.24 (0.44-11.52) | 0.83 (0.68-1.01) | 2.05 ( 1.12-3.74) | 1.87 (1.07-3.28) |
| Modena | 1.25 (0.78-2.00) | 4.65 (1.07-20.08) | 0.42 (0.34-0.51) | 1.49 (0.83-2.68) | 2.48 (1.48-4.16) |
| Pavia | 1.57 (1.01-2.45) | 3.36 (0.77-14.59) | 0.78 (0.65-0.93) | 2.34 (1.35-4.06) | 1.62 (0.96-2.73) |
| Ragusa-Caltanissetta | 1.63 (1.01-2.62) | 0.75 (0.11-5.30) | 0.90 (0.75-1.09) | 1.56 (0.85-2.88) | 1.43 (0.81-2.53) |
| Sondrio | 1.42 (0.85-2.36) | 3.85 (0.82-18.13) | 0.80 (0.65-0.98) | 1.20 (0.61-2.36) | 1.74 (0.98-3.09) |
| Aosta Valley | 1.35 (0.72-2.54) | 10.43 (2.29-47.55) | 0.66 (0.50-0.89) | 1.16 (0.49-2.75) | 1.56 (0.76-3.20) |
| Trapani | 1 | 1 | 1 | 1 | 1 |

***** Fine-Gray model stratified by age class
